# Supplementary material for: Peroxisomal Import Stress Drives Cellular Senescence through SCAF1-dependent Suppression of Mitoribosome Biogenesis
Source: bioRxiv. 2026 Sep 23:2026.09.19.752713. Preprint. [Version 1] doi: 10.64898/2026.09.19.752713 (PMC13622025; doi:10.64898/2026.09.19.752713)
Supplement: Supplementary file 1 [file NIHPP2026.09.19.752713v1-supplement-1.pdf]

bioRxiv preprint doi: https://doi.org/10.64898/2026.09.19.752713; this version posted September 23, 2026. The copyright holder for this preprint (which was not certified by peer review) is the author/funder, who has granted bioRxiv a license to display the preprint in perpetuity. It is made available under a CC-BY 4.0 International license.

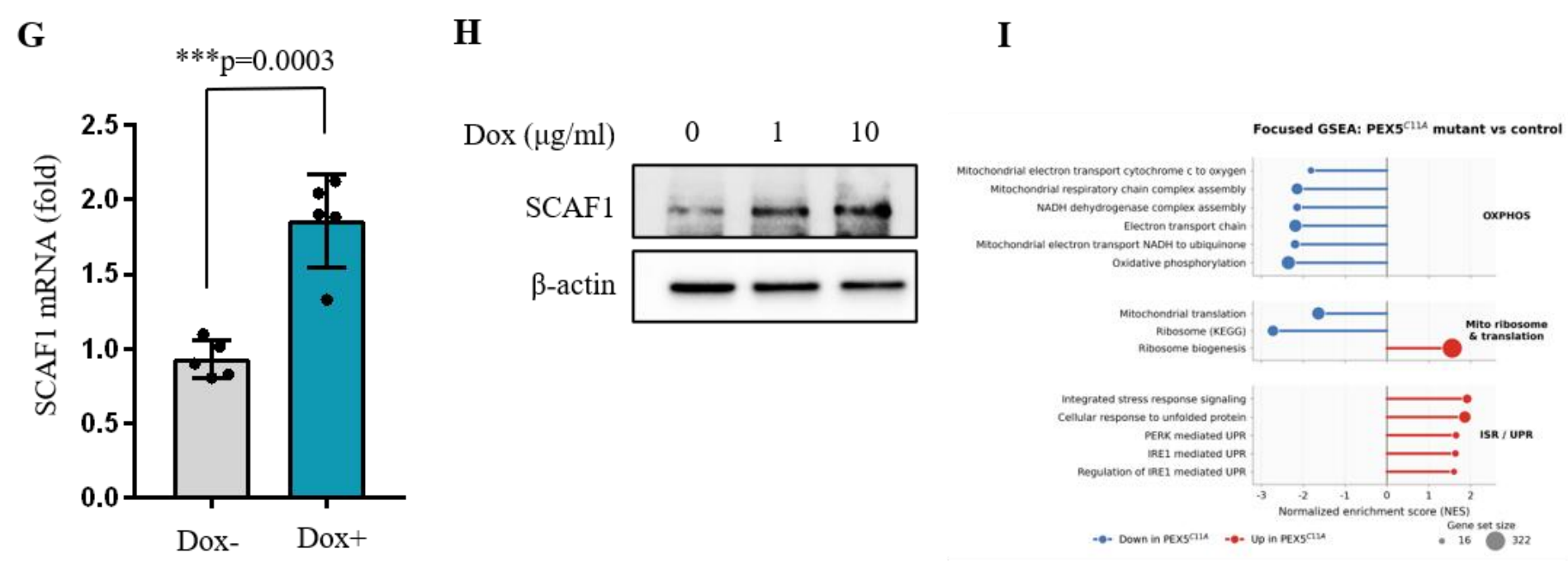

Supplementary Fig. 1. CRISPR screen identify SCAF1 as a regulator mediating peroxisome stress (G) SCAF1 mRNA expression was measured by qPCR after 3 days of doxycycline treatment (1µg/ml) on Tet-On PEX5^{C11A} cells. ***p =0.0003, mean ± SD, t-test. (H) SCAF1 protein expression was measured by western blotting after 3 days of doxycycline treatment (0, 1, or 10 µg/ml) on Tet-ON PEX5^{C11A} cells . (I). Gene set Enrichment Analysis of Mitochondrial and stress response pathways in Tet-On PEX5^{C11A} cells. Bar chart showing normalized enrichment scores (NES) for significantly enriched gene sets (padj<0.05) across KEGG , HALLMARK and GO Biological Process (GO:BP) databases . Bars extending left (blue) indicate downregulation in PEX5 ^{C11A} mutant relative to control; bars extending right (red) indicate upregulation. GSEA was performed using the fgsea package in R with gene sets from MSigDB.
